# Possible mechanisms of CO_2_ reduction by H_2_ via prebiotic vectorial electrochemistry

**DOI:** 10.1098/rsfs.2019.0073

**Published:** 2019-10-18

**Authors:** Rafaela Vasiliadou, Nikolay Dimov, Nicolas Szita, Sean F. Jordan, Nick Lane

**Affiliations:** 1 Centre for Life's Origin and Evolution, Department of Genetics, Evolution and Environment, University College London, Darwin Building, Gower Street, London WC1E 6BT, UK; 2 School of Engineering and Computer Science, University of Hertfordshire, College Lane, Hatfield AL10 9AB, UK; 3 Department of Biochemical Engineering, University College London, Bernard Katz Building, Gower Street, London WC1E 6BT, UK

**Keywords:** CO_2_ reduction, origin of life, vectorial chemistry, energy-converting hydrogenase, alkaline hydrothermal vents, microfluidic reactor

## Abstract

Methanogens are putatively ancestral autotrophs that reduce CO_2_ with H_2_ to form biomass using a membrane-bound, proton-motive Fe(Ni)S protein called the energy-converting hydrogenase (Ech). At the origin of life, geologically sustained H^+^ gradients across inorganic barriers containing Fe(Ni)S minerals could theoretically have driven CO_2_ reduction by H_2_ through vectorial chemistry in a similar way to Ech. pH modulation of the redox potentials of H_2_, CO_2_ and Fe(Ni)S minerals could in principle enable an otherwise endergonic reaction. Here, we analyse whether vectorial electrochemistry can facilitate the reduction of CO_2_ by H_2_ under alkaline hydrothermal conditions using a microfluidic reactor. We present pilot data showing that steep pH gradients of approximately 5 pH units can be sustained over greater than 5 h across Fe(Ni)S barriers, with H^+^-flux across the barrier about two million-fold faster than OH^–^-flux. This high flux produces a calculated 3-pH unit-gradient (equating to 180 mV) across single approximately 25-nm Fe(Ni)S nanocrystals, which is close to that required to reduce CO_2_. However, the poor solubility of H_2_ at atmospheric pressure limits CO_2_ reduction by H_2_, explaining why organic synthesis has so far proved elusive in our reactor. Higher H_2_ concentration will be needed in future to facilitate CO_2_ reduction through prebiotic vectorial electrochemistry.

## 1. Life as a guide to prebiotic chemistry

The origin of life has not been considered a question in biology until recently—prebiotic chemistry, by definition, took place before biology began. This perspective is now changing. Phylogenetics has restructured the deepest branching in the tree of life [1–6], while comparative physiology has uncovered revelatory new mechanisms of anaerobic metabolism, notably the process of flavin-based electron bifurcation [7–10]. These findings suggest that the last universal common ancestor (LUCA) was, in fact, the ancestor of bacteria and archaea [1,2,11], the eukaryotes being a derived domain [12]. The deepest branches in these groups suggest that LUCA was an obligately chemiosmotic autotroph that grew from CO_2_ and H_2_ by some form of the acetyl CoA pathway [1,13–15], extending into the formation of C2–C5 carboxylic acids, notably Krebs cycle intermediates [16–18]. We have previously argued that the metabolism of LUCA was probably similar to CO_2_ fixation in modern methanogens [19,20]; the notion that methanogenesis is ancient is itself a venerable idea, dating back to their discovery [21,22].

While there is no necessary link between LUCA and prebiotic chemistry, neither is there necessarily no link. Most prebiotic chemistry over decades has focused on relatively facile reactions involving reactive precursors such as cyanoacetylene activated by UV radiation [23,24]. While impressive chemistry, successfully producing informational monomers such as activated pyrimidine nucleotides [23], this ‘cyanosulfidic protometabolism' bears little resemblance to the biochemistry of known cells in terms of substrates, pathways, catalysts or energy coupling [25]. By contrast, recent work shows that strong electron donors such as native iron [26,27], or 1 V electrical potential [28], can reduce CO_2_ to carboxylic acids, including all five universal intermediates in life's core metabolism—acetate, pyruvate, oxaloacetate, succinate and α-ketoglutarate [26,27,29,30]. Intriguingly, glyoxylate can drive interconversions between all Krebs cycle intermediates [29]. These findings are an important proof of concept and link beautifully with the structure of metabolism, in which the hydrogenation of CO_2_ forms primarily C2–C5 carboxylic acids—carbon skeletons—from which are formed amino acids, fatty acids, sugars and eventually nucleotides [16–18,25,26,31]. Amino acids [29,32], fatty acids [33] and sugars [34,35] have been synthesized from carboxylic acids or their derivatives under equivalent prebiotic conditions, though nucleotides have proved more difficult so far [25]. Many of these reaction pathways were proposed to occur spontaneously in alkaline hydrothermal vents by Martin and Russell more than a decade ago [18,36] and these recent experimental findings confirm their predictions.

But there are also some issues with the use of native iron as an electron donor for CO_2_ fixation, or indeed any larger-scale mineral surface as a source of organic precursors. By larger scale, we mean any mineral assemblage that could not be readily inherited by daughter cells. Life requires genetic information, and prebiotic systems must obviously be capable of giving rise, through a continuous (and testable) set of steps, to the origins of the genetic code and natural selection. Genetic heredity is a specific form of growth—for RNA or DNA to be copied, one template must give rise to two, and so on, equating to growth and the formation of nucleotides. From a thermodynamic point of view, it is easier to make carboxylic acids, amino acids, fatty acids and sugars than nucleotides (which have still not been synthesized through prebiotic reactions that resemble biochemistry [25]). This means that any ‘RNA world' is necessarily dirty, contaminated with other organic molecules [37–41]. So, the question becomes: how can a growing, replicating system get better at making copies of itself, with increasingly complex prebiotic chemistry eventually giving rise to nucleotides and genetic information? This question is much easier to answer if the catalysts that drive growth (CO_2_ fixation) are inherited by daughter cells [42]. That, in turn, makes sense of the known process of CO_2_ fixation in anaerobic prokaryotes, which use inorganic mineral-like structures such as Fe(Ni)S clusters to fix CO_2_. These clusters are small, can self-assemble from ions in solution (through chelation) [43,44], can be oxidized and reduced in turn [45], and may be inherited directly, for example, in association with cell membranes [42]. We have shown that FeS clusters (including 4Fe–4S and 2Fe–2S clusters) can form spontaneously under alkaline conditions through interactions between Fe^2+^, Fe^3+^, S^2–^ and amino acids such as cysteine, demonstrating a potential path to biological FeS clusters [46]. A larger-scale mineral, or even nanoparticles of native iron, could not be inherited in this way, hence do not point to a clear path from prebiotic chemistry to genetic heredity.

**Figure 1.**
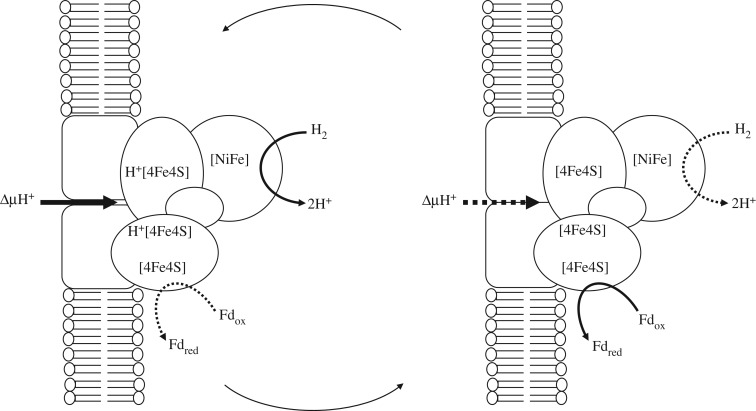
Possible mechanism of the energy-converting hydrogenase (Ech). Ech reduces ferredoxin (Fd) with electrons from H_2_, despite a 200 mV difference in redox potential in methanogens [41]. (*a*) H^+^ pore is open and H^+^ binds to ligands associated with the two pH-modulated 4Fe–4S clusters, raising the redox potential of all four clusters through electron delocalization via quantum tunnelling and so enabling their reduction by H_2_. (*b*) H^+^ pore closes and H^+^ dissociates from ligands, lowering the redox potential of the final 4Fe–4S cluster sufficiently to reduce Fd_ox_ to Fd_red_.

The key enzyme required to reduce CO_2_ to biomass in methanogens is the energy-converting hydrogenase (Ech) [45,47,48]. This is a membrane-bound, proton-motive Fe(Ni)S protein that uses H_2_ to reduce a low-potential ferredoxin directly [45,48,49]. The critical point here is that, even though this reduction is not favoured under standard conditions, the inward flux of protons through Ech can overcome an endergonic barrier of around 40 kJ mol^−1^, equating to a difference in redox potential of 200 mV, given an H_2_ partial pressure of 10 Pa and an oxidized-to-reduced ferredoxin ratio (Fd_ox_/Fd_red_) of less than 0.01 [47]. The mechanism by which Ech reduces ferredoxin is not known, but two of the four Fe(Ni)S clusters have a pH-dependent reduction potential, becoming more reducing by –50 mV per pH unit, in rough accordance with the Nernst equation [49,50]. Given their close proximity to the transmembrane proton-pore [51], it seems plausible that these two FeS clusters could be reduced by H_2_ only when protonated, and could reduce ferredoxin only when deprotonated (figure 1). This putative mechanism is of major relevance to the origin of life because it implies that the most basic requirement to reduce CO_2_ using H_2_ is a dynamic proton flux across Fe(Ni)S clusters.

We have previously shown that hydrothermally sustained proton gradients in alkaline vents can in principle promote the reduction of ferredoxin by H_2_ via Ech in the absence of any active pumping [52]. The continuous flux of protons into simple cells down a hydrothermal gradient can be maintained indefinitely, so long as their membranes are as permeable to protons as fatty-acid vesicles (which allows the escape or neutralization of protons) [52,53]. More pertinently here, prebiotic homologues of Ech with Fe(Ni)S clusters chelated by short polypeptides [43] or even amino acids [44,46] could associate with fatty-acid membranes to drive CO_2_ fixation in geochemical proton gradients [42]. Computer simulations show that positive feedbacks through physical interactions between dissolved ions, amino acids and fatty-acid bilayers can, in fact, promote rudimentary membrane heredity [42]. These positive feedbacks mean that the more organics that are formed, the more Fe(Ni)S clusters are chelated, and the more likely they are to associate with the membrane, driving synthesis of more organics as a ‘proto-Ech'. Such clusters are homologous with living systems [3,14], form spontaneously inside protocells [43,44], are heritable and evolvable [42]—for example, being chelated initially by amino acids [44,46], later by short non-coded polypeptide nests [54], and ultimately genetically encoded proteins [55]—can be oxidized and reduced in turn [20,47], and confer a direct selective advantage to protocells even before the emergence of genetic heredity [42].

Yet while Fe(Ni)S clusters chelated by amino acids and associated with fatty-acid membranes could in principle power protocell growth and evolution through a series of continuously connected steps, they are already quite sophisticated organic systems. How did such systems arise? Their reliance on mineral-like Fe(Ni)S clusters, hydrothermally sustained proton gradients and the abundant gases H_2_ and CO_2_, which both have pH-dependent reduction potentials, implies that a wholly inorganic form of vectorial electrochemistry could have driven CO_2_ fixation to generate organics at the origin of life [13,18,36,40,41,53,56–58].

## 2. Vectorial electrochemistry at the origin of life

If the arguments in the previous section are correct then the requirements for prebiotic CO_2_ fixation by H_2_ would be steep pH gradients across thin inorganic barriers containing Fe(Ni)S minerals, with a physical topology resembling that of cells [41,59]. This idea is not new, but exactly how geochemical proton gradients could facilitate the reduction of CO_2_ has been the subject of debate [53,60]. We have previously argued that the key feature is the pH-dependent reduction potentials of the reactants and catalysts — H_2_, CO_2_ and Fe(Ni)S minerals [19,20,58,61,62].

There are two cardinal factors here. First, the redox potential of H_2_ is not low enough to reduce CO_2_ at any equivalent pH, hence the difficulty of doing so in solution by scalar chemistry under prebiotic conditions. However, if H_2_ is dissolved in solution at a substantially higher pH than CO_2_, which is the case in the physically structured environment of alkaline hydrothermal vents, then in principle this reaction could proceed. Second, FeS clusters transfer single electrons rather than pairs of electrons, hence even if they are strongly reducing, they are unlikely to reduce CO_2_ directly [63]. One potential solution is also offered by cells: molybdenum (Mo) can receive electrons from FeS clusters and pass them on, effectively in pairs, to CO_2_ [41]. As in the Fe(Ni)S clusters of Ech, FeS minerals could then be reduced from Fe^3+^ to Fe^2+^ by H_2_ when protonated, and reduce Mo^6+^ to Mo^4+^ when deprotonated [41,64]. Mo^4+^ could, in turn, reduce CO_2_ to form organics [41]. Mo^6+^ is soluble in alkaline solution, hence could co-assemble with FeS minerals when oxidized by CO_2_ in alkaline hydrothermal conditions [41]. Mo is also notable in that its redox potential is pH-dependent [65], and displays crossed-over electron bifurcation dynamics, making it capable of driving very low-potential reductions [41,64].

The reason that pH differences across inorganic barriers could facilitate the reduction of CO_2_ is that the redox potential of the H_2_/2H^+^ couple falls by about –58 mV per pH unit, according to the Nernst equation, from –414 mV at pH 7 to –646 mV at pH 11 [66]. By contrast, the redox potential of the CH_2_O/HCOO^–^ couple (the most endergonic step of CO_2_ reduction) rises from –580 mV at pH 7 to –522 mV at pH 6 [58], reflecting the likely pH of the Hadean oceans [67–70]. This means that the first steps in the reduction of CO_2_ by H_2_ are strongly endergonic if the pH is equivalent (+38 kJ mol^−1^) but become moderately exergonic when the pH of the reactants differs in a structured environment (–18 kJ mol^−1^). A difference of 5 pH units, therefore, confers a substantial free energy change of –56 kJ mol^−1^. Indeed, the redox potential of H_2_ in strongly alkaline conditions is low enough to reduce Fe^2+^ to Fe^0^, possibly enabling the formation of direct metal–carbon bonds as in some ancient metalloenzymes including [Fe–Ni] hydrogenase and radical SAM [63]. But these values assume a standard partial pressure of H_2_, giving a saturation value of about 0.8 mM at atmospheric pressure, as well as a very steep gradient of 5 pH units across a distance of just a few nanometres neither of which conditions might be met in abiotic compartments compared with cells.

That brings us to the crucial open question about how closely a structured inorganic environment could resemble the topology of cells. Ech is a membrane-bound Fe(Ni)S protein spanning a bilayer just 5 nm in diameter [5,44,47,48]. The FeS clusters are closely juxtaposed in space next to the transmembrane proton pore [51]. Presumably conformational changes in the transmembrane domains facilitate proton transfer (or Ech would not be able to function in reverse as a proton pump, powered by ferredoxin oxidation) [47,48]. This means that the FeS clusters should be subject to an oscillating flux of protons, with protons binding to FeS clusters or their ligands when the membrane pore is open (figure 1*a*) and then detaching and entering the cell when the pore is closed (figure 1*b*). By contrast, the inorganic barriers separating channels or pores in alkaline hydrothermal vents may be many micrometres thick [62,71–73], with nothing clearly resembling an oscillating flux of protons focused through membrane pores on a nanometre scale. It's also worth noting that the concept of pH refers to proton concentration in bulk solution and has little meaning in confined spaces. So the presence or absence of a single proton in a transmembrane channel would imply a binary switch from strong acidity to strong alkalinity, potentially much greater than 5 pH units, and therefore potentially able to drive difficult reactions with some ease [74]. This could not occur with any mixing in bulk solution, and arguably not even with unfocused transfer of protons across barriers. Given this disparity, could a broad topological equivalence hold at the nanoscale, driving CO_2_ fixation across inorganic barriers?

**Figure 2.**
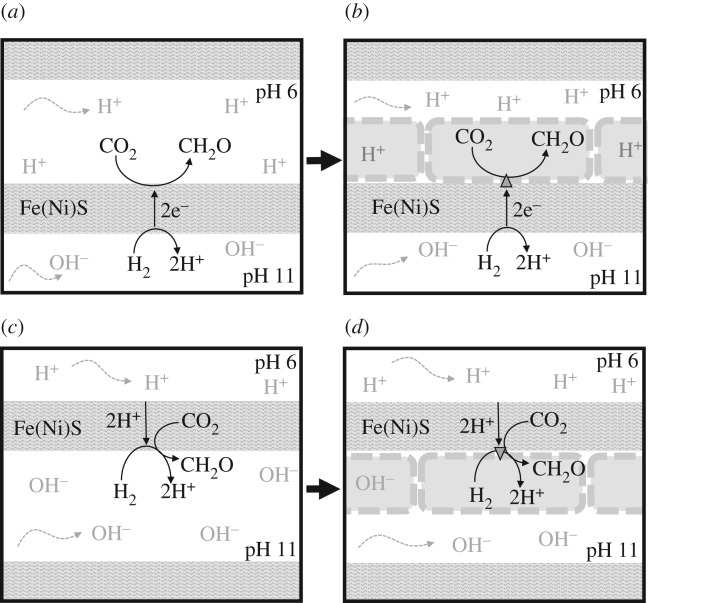
Topology of hypothetical vectorial chemistry across an inorganic barrier containing Fe(Ni)S nanocrystals. (*a*) Transfer of electrons (e^–^) across the whole barrier from H_2_ in alkaline solution (which lowers H_2_ redox potential) to CO_2_ in acidic ocean water (which raises its redox potential). (*b*) Topology of CO_2_ reduction in protocells at a later stage assuming continuity of CO_2_ reduction with (*a*). The triangle depicts Fe(Ni)S clusters in a membrane-bound proto-Ech. Note this is not topologically equivalent to CO_2_ reduction via Ech in modern cells. (*c*) Transfer of H^+^ across the whole barrier. CO_2_ reduction could hypothetically occur in a narrow region with sharp pH gradients across single 20 nm mackinawite nanocrystals close to the alkaline face. (*d*) Topology of CO_2_ reduction in protocells at a later stage assuming continuity of CO_2_ reduction with (*c*). Note that this *is* topologically equivalent to CO_2_ reduction via Ech in modern cells.

There are two possible ways in which the analogy could hold at this nano-level. First, FeS minerals such as mackinawite are semiconducting (in the longitudinal plane only) and so could theoretically transfer electrons from H_2_ in alkaline solution on the inside of a pore, to CO_2_ in acidic ocean water outside [53,62,75,76]. If so, then CO_2_ reduction would take place in the acidic ocean phase (figure 2*a*). That is not to say that organic molecules would be lost to the ocean [77]; rather, ocean waters should percolate into the labyrinth of pores inside the vent [78] and potentially even concentrate organics by thermophoresis [79,80]. But there are other issues too. Is it feasible for electrons to conduct across these relatively thick barriers, given that mackinawite only conducts in one plane [81,82]? Evidence from black-smoker vents (which are much more metal-rich, so probably better conductors) suggests that electrons can indeed conduct over several centimetres [83], but experimental work on hollow FeS vent-like structures in the laboratory suggests that they typically retain an electrical potential difference over several hours [84], implying that they do not conduct electrons readily.

A subtler question concerns the topology, which in this case is not equivalent to that of cells. With Ech, the electrons derive from H_2_, which is dissolved in the relatively alkaline interior of the cell, whereas the reduction of CO_2_ is facilitated by an influx of protons from the more acidic exterior [47]. If electrons were transferred across a barrier, CO_2_ reduction would take place on the acidic exterior (figure 2*a*). The primary vectorial flux would then be electron flow, not proton flow. That would imply a discontinuity between prebiotic vectorial chemistry and the origins of biochemical CO_2_ reduction via Ech: the electrons for CO_2_ reduction derive from Fe^2+^ in the barrier outside the protocell, rather than from H_2_ inside the cell, and there is no primary role for a transmembrane proton gradient. A second difficulty with this hypothesis is that, at a later stage (figure 2*b*), protocells would eventually form on the acidic face of the barrier. However, bilayer membranes cannot self-assemble from single-chain amphiphiles (e.g. fatty acids and fatty alcohols) at even mildly acidic pH (approx. 6), so simple protocells depicted in figure 2*b* could only form if negatively charged head-groups are condensed onto the fatty acids, a more complex and therefore less likely scenario [85–87]. It is also possible that organics could form in the acidic phase and then accumulate by thermophoresis elsewhere in the system [79,80]. However, that too implies a discontinuity between organic synthesis and protocell formation which is at the least convoluted. We, therefore, do not favour this possibility in principle, despite having advocated it in the past [53,61,62].

The second possible way in which the analogy could hold at a nano-level would be for protons to transfer across the barrier rather than electrons. Transfer of protons rather than electrons across a barrier is more strictly analogous to the topology of the Ech, as organics would now be formed from H_2_ in the alkaline phase, with the flux of H^+^ across the barrier modulating the redox potential of H_2_, CO_2_ and Fe(Ni)S minerals on the alkaline side. For this scenario to work, however, very steep pH gradients would be required on the alkaline side of the barrier. If electrons could conduct across single nanocrystals but not across multiple crystals with scattered orientation, then these steep pH gradients would need to develop across single Fe(Ni)S nanocrystals, of approximately 20–30 nm in length. When protonated, the Fe(Ni)S nanocrystal would be reducible by H_2_ on its alkaline side (where the redox potential for H_2_/H^+^ couple would potentially be close to –650 mV). Conversely, when deprotonated, its redox potential would fall low enough to reduce CO_2_ on the acidic side of the nanocrystal (where the reduction potential for the CO_2_/CH_2_O couple could be closer to –520 mV). If that were the case, then alternating protonation and deprotonation of Fe(Ni)S nanocrystals would potentially facilitate CO_2_ reduction in a similar fashion to the Ech. By modulating the redox potential of Fe(Ni)S nanocrystals, H_2_ and CO_2_, sharp differences in pH alter the ΔG of the reaction, in other words, the thermodynamic driving force. That requirement, in turn, demands that H^+^ should transfer across the barrier much more rapidly than OH^–^ from the alkaline side, otherwise H^+^ and OH^–^ would neutralize within the barrier, dissipating any steep pH gradients and ruling out this postulated mechanism.

Whether it is really feasible for H^+^ to cross inorganic barriers in hydrothermal vents so much more easily than OH^–^ is unknown. The conductance of H^+^ through bulk water is less than double that of OH^–^ [88]. Unlike OH^–^, however, it is known that protons can conduct rapidly through intercalated nanoconfined water in layered minerals, including mackinawite, via the Grotthuss mechanism that takes place in bulk water and protein cavities [89–91]. How much faster H^+^ transfer might be across freshly precipitated, unmineralized barriers with a high aqueous content—likely to characterize the active regions of hydrothermal systems discussed here—remains an open question that has been debated in the literature [53] with some arguing that such steep pH gradients are not feasible in hydrothermal systems [60]. On the other hand, if the difference in mobility is substantial, then fast conductance of protons could foreshadow the mechanism of Ech in a prebiotic setting, with electron conductance taking place locally across nanocrystals rather than across the entire barrier (figure 2*c*). If that were the case, then the topology of protocells would be correct (figure 2*d*). We have previously shown that vesicles formed from single-chain amphiphiles such as fatty acids and fatty alcohols are stable at pH 11 and above [92,93], so the scheme depicted in figure 2*d* is feasible. The major question that we explore now is whether suitably steep H^+^ gradients could develop across inorganic barriers containing disordered Fe(Ni)S minerals, which could ultimately promote the reduction of CO_2_ by H_2_.

## 3. A microfluidic chip to simulate prebiotic vectorial electrochemistry

**Figure 3.**
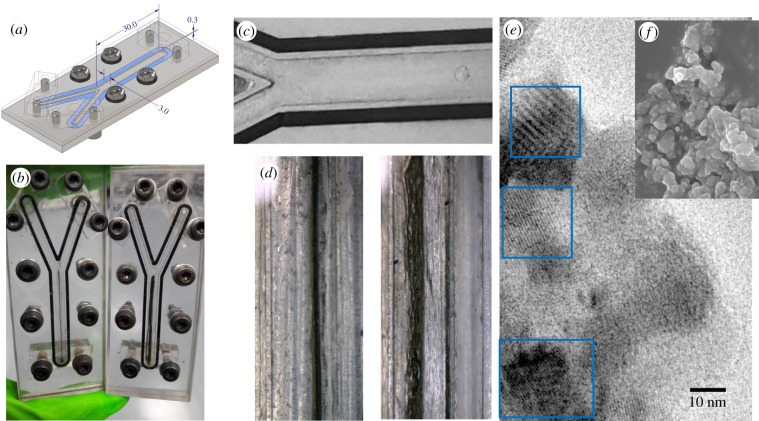
Design and function of microfluidic reactor. (*a*) Design of reactor showing Y-shaped channel. Dimensions in millimetres. (*b*) Two microfluidic reactors showing fresh precipitate after 45 min (left) and 5 h (right). (*c*) Confluence of Y-shaped channels giving parallel laminar flow down main channel. (*d*) Magnification of main channel showing Fe(Ni)S precipitate after 1 h (left) and 5 h (right), showing lacy network structure. (*e*) Transmission electron micrograph of barrier showing lattice structure of Fe(Ni)S nanocrystals; note the disordered orientation. (*f*) Scanning electron micrograph showing microgeodes composed of mixed Fe minerals. (Online version in colour.)

We have fabricated a microfluidic reactor to test these questions by simulating the flow dynamics of anoxic Hadean alkaline hydrothermal vents. The chip design is shown in figure 3*a–c*. Reactors were made from aluminium or polycarbonate depending on experimental requirements. All experiments were carried out in an anaerobic hood in an atmosphere of 5% H_2_∶95% N_2_. Alkaline hydrothermal fluids were simulated as a solution of sodium sulfide (NaS, 1 mM) and sodium silicate (Na_6_Si_2_O_7_, 10 mM). This solution was adjusted to pH 11 using NaOH as needed. H_2_ gas was introduced into the alkaline solution via a T-connector from a syringe containing aluminium powder and 1 M NaOH, replenished every 30 min. Hadean oceans were simulated as a solution of ferrous chloride (FeCl_2_, 5 mM), nickel chloride (NiCl_2_, 1 mM) and sodium bicarbonate (NaHCO_3_, 10 mM) [62,72,73]. The ocean solution was adjusted to pH 6 using HCl, this acidity reflecting likely Hadean conditions (when the atmospheric CO_2_ levels were at least 100-fold higher) [67–70] and favour a 50 : 50 partitioning of CO_2_ and $\mathrm{HC}O_{3}^{\text{–}}$. While CO_2_ is most likely the species reduced [94] $\mathrm{HC}O_{3}^{\text{–}}$ is also possible [28]; the pH used should permit either reaction. Small bubbles of H_2_ could be seen in the alkaline solution, implying a concentration close to saturation at atmospheric pressure. Occasionally, small bubbles of CO_2_ could be observed in the acid ocean, showing partitioning from $\mathrm{HC}O_{3}^{\text{–}}$ to CO_2_ at pH 6. These bubbles mostly progressed through the chip and rarely obstructed flow. With the exception of $\mathrm{HC}O_{3}^{\text{–}}$, which was introduced as a substrate into the acid channel only, we avoided the use of buffers as our intention was to simulate prebiotic hydrothermal environments which are not buffered.

The solutions of alkaline hydrothermal fluids and Hadean ocean were introduced through a Y-shaped channel on the chip at optimal flow rates of 50 µl min^−1^, giving parallel laminar flow through the main channel of the chip. The entire chip was heated to 70°C to simulate the temperature of vent fluids, which should favour the reaction between H_2_ and CO_2_ thermodynamically. A thin, stable precipitate of Fe(Ni)S minerals including mackinawite nanocrystals formed spontaneously within approximately 45 min, and thickened slowly over 5 h to fill approximately 50% of the channel (figure 3*d*). Mackinawite nanocrystals had been identified and characterized in earlier work [62] and were equivalent in structure here, with individual nanocrystals being 20–30 nm in length and approximately 10 nm in width, with spacing between the atomic planes of 0.3 nm, 0.5 nm and 0.5 nm for the g, h and i planes, respectively (figure 3*e*). High densities of these nanocrystals were observed, but with scattered orientation. This disordered orientation is unlikely to promote the transfer of electrons across the barrier, given that mackinawite is semiconducting in longitudinal plane only. That interpretation is supported by scanning electron micrographs, which show both wall structures and microgeodes composed of mixed ferrous hydroxides, carbonates, sulfides and silicates (figure 3*f*); there is no regular structure that would clearly promote electrical conductance. These findings are in accordance with earlier work showing the retention of an electrical potential difference over several hours [84].

Most experiments on the microfluidic chip were terminated after 5–6 h, but replacing the solutions with equivalent solutions at the same pH but lacking NaS, FeCl_2_ and NiCl_2_ (so no longer thickening the precipitate) enabled flow to be continued over 24 h without blocking the channel or causing back-pressure problems. The chip was designed with a single outflow channel, meaning that there was mixing of alkaline and acid solutions in the effluent. The rationale for mixing the effluent related to the transfer of electrical charge across the barrier, regardless of whether electrons or protons are transferred. Any charge on the barrier would oppose continued electrical flux unless there is a salt bridge (as a transfer of counter-ions across the barrier) or mixing elsewhere in the system. Mixing certainly takes place in vents, and in principle should dissipate any electrical charge. The mixing of effluent from the chip should, therefore, promote a continuous transfer of charge across the barrier in either direction, potentially facilitating CO_2_ reduction even if counter-ions crossed the barrier far more tardily.

**Figure 4.**
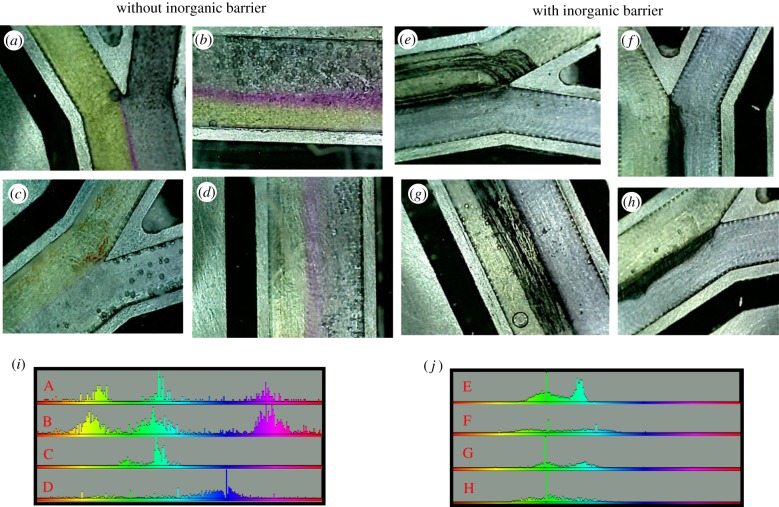
pH gradients sustained by laminar flow (50 µl min^−1^) on the microfluidic reactor in the absence (*a*–*d*) and presence (*e*–*h*) of freshly precipitated inorganic Fe(Ni)S barriers. pH-sensitive dyes used were phenol red (acidic channel, yellow green) and universal indicator (alkaline channel, pale blue). The colour bars (*i*,*j*) show the colour balance across the entire channel generated by ImageJ software. In the absence of a barrier (*a*–*d*), the red, dark blue, yellow and pink colours show mixing between the two flows. In the presence of barriers (*e*–*h*), there are only two colours (green and pale blue) corresponding to the acidic and alkaline channels with little if any mixing. Note that the barriers in (*e*) and (*g*) are lacy network precipitates with aqueous channels that are clearly acidic pH.

**Figure 5.**
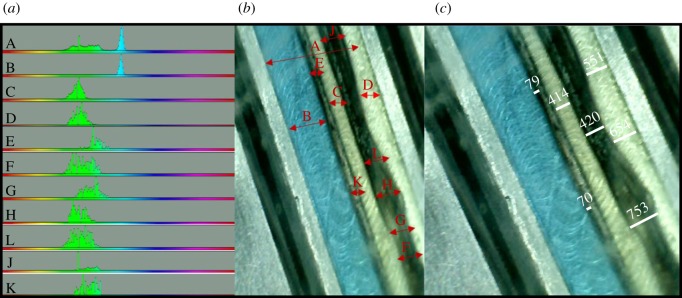
Differences in pH between the acidic and alkaline channels, and aqueous channels trapped within the barrier. The dyes are phenol red (acid channel) and bromothymol blue (alkaline channel). (*a*) Shows the colour balance across the regions delineated in (*b*). There is clearly no mixing of acidic and alkaline fluids in aqueous channels within the barrier, which all retain a pH of approximately 6 (yellow-green colour). (*c*) Shows distances marked on the same image, with a minimal distance of 70 µm between acidic aqueous channels within the barrier and the alkaline channel. This image is representative of *N* = 6 equivalent runs with lacy network precipitates.

A flow rate of 50 µl min^−1^ can sustain laminar flow and visible pH gradients even in the absence of a barrier (using acidic and alkaline solutions lacking NaS, FeCl_2_ and NiCl_2_; figure 4*a*–*d*). This difference in pH can be maintained over 24 h in the presence of thin Fe(Ni)S barriers. With a continuous flux of solutions containing NaS, FeCl_2_ and NiCl_2_, the barrier thickened gradually over 5 h, usually (but not invariably) on the alkaline side. The Fe(Ni)S barriers tend to form lacy network structures frequently containing elongated aqueous channels within the barrier (figure 4*e,g*) but are occasionally dense (figure 4*f*,*h*). Inclusion of pH-sensitive dyes in the solutions (phenol red for acid ocean and bromothymol blue or universal indicator for alkaline fluids) showed that these aqueous inclusions within network barriers were almost always acidic, giving a yellow-green colour with phenol red and bromothymol blue (figure 5*a*). Note that at 70°C, K_w_ = 7.16 × 10^–14^, so the [H^+^] and [OH^–^] at neutral pH = 2.77 × 10^–7^, giving a neutral pH of 6.57. Because the alkaline fluids are pH 11 (giving an [OH^–^] of 2.77 × 10^–3^ M at 70°C) and acidic oceans are pH 6 (giving an [H^+^] of 2.77 × 10^–6^ M at 70°C), the concentration of OH^–^ ions is 1000-fold greater than H^+^ ions. Despite this difference, the fact that the barrier becomes acidic in virtually all cases implies that H^+^ transfer across the barrier is much faster than OH^–^ transfer (figures 4*e*,*g* and 5*b*). The faster transport of H^+^ across the barrier gives steep pH gradients, but we could only measure these directly between aqueous inclusions in the barrier and the alkaline channel, with the steepest measurable gradients being approximately 5 pH units across approximately 70 µm (figure 5*c*). However, this is still roughly 3500-fold greater than the approximately 20 nm length of individual Fe(Ni)S nanocrystals, across which pH gradients would arguably need to operate to facilitate CO_2_ reduction by vectorial chemistry under strictly prebiotic conditions. Could pH gradients be so much steeper within the precipitates?

To estimate the distance across which proton gradients might in fact operate, we stopped the flow in the acidic and alkaline channels in turn to calculate the permeability of the barrier to H^+^ and OH^–^, respectively. These are approximate order of magnitude calculations. After stopping the flow in the alkaline but not the acidic channel, we measured the time taken for half of the alkaline channel to become acidic (pH 6), meaning a colour change from blue to yellow-green. The mean time for half the alkaline channel to become acidic (pH 6) was 2.7 ± 1.5 s (*N* = 3). The volume of the chip = 3 × 10^–5^ l, so half the volume of the alkaline channel alone is 7.5 × 10^–6^ l. The pH changed from approximately 11 to 6 in half the alkaline channel. The concentration of OH^–^ at pH 11 = 2.77 × 10^–3^ M. That means the number of OH^–^ ions neutralized in 2.66 s = 6.023 × 10^23^ (Avogadro's number) × 0.00277 ([OH^–^]) × 7.5 × 10^–6^ (acid volume) = 1.3 × 10^16^ OH^–^ ions neutralized in 2.7 s, or 4.6 × 10^15^ H^+^ crossed the barrier in 1 s. The permeability of the barrier to protons = P = J/AC, where J = flux, A = area of precipitate and C = [H^+^]. Therefore, *p* = 4.6 × 10^15^/4.5 × 10^–5^ m^2^ × 2.77 × 10^–6^ M protons (pH 6) = 3.7 × 10^25^ H^+^/s.

For the permeability of the barrier to OH^–^ ions, the pH changed from 6 (yellow-green) to 8 (blue) in 5.0 ± 2.0 s (*N* = 3). Thus, 2.77 × 10^–6^ M protons were neutralized in a volume of 7.5 × 10^–6^ l, or a total of 1.3 × 10^13^ H^+^ were neutralized in 5 s = 2.5 × 10^12^ OH^–^/s. The permeability of the barrier to OH^–^ = 2.5 × 10^12^ (flux)/4.5 × 10^–5^ (area of barrier) × 0.00277 (concentration of OH^–^ at pH 11) = 2.01 × 10^19^ OH^–^/s. This means the permeability of the barrier to H^+^ is 1.84 million-fold greater than the permeability to OH^–^, or approximately two million-fold more permeable to H^+^, allowing for the uncertainty around several of these measurements. Nonetheless, this value is clearly roughly correct given that aqueous channels within the barrier are acidic despite (i) the [OH^–^] is 1000-fold greater than the [H^+^] (pH 11 versus pH 6); (ii) the shift in pH in the alkaline channel is from pH 11 to 6 (blue to yellow-green, from a known pH of 11 to a measured pH of 6), meaning 10^–3^ M OH^–^ solution is neutralized, whereas the pH shift in the acidic channel is from pH 6 to 8 (because bromothymol blue turns blue at pH 8), meaning just 10^–6^ M H^+^ solution is neutralized; and (iii) the times for H^+^ to transfer across the barrier were about twice those for OH^–^. So we are satisfied that the permeability of the Fe(Ni)S barrier to H^+^ is about two million-fold greater than OH^–^. This is consistent with the premise that H^+^ is transferred through intercalated, nanoconfined water in layered minerals like mackinawite (and fresh precipitates) via a Grotthuss mechanism, whereas OH^–^ ions cannot [89–91].

The conclusion that H^+^ ions transfer across Fe(Ni)S barriers about two million-fold faster than OH^–^ ions permits us to roughly constrain the likely steepness of the H^+^ gradient across the approximately 70 µm precipitate that was the steepest experimentally measurable gradient (5 pH units). Because [OH^–^] in the alkaline channel is 1000-fold greater than [H^+^] in the acidic channel, the difference in the rate of ions crossing the barrier should be approximately 2000-fold. That means there should be a point at which the transfer of OH^–^ ≈ H^+^ at 1/2000 of the distance from the alkaline channel to the acidic inclusion, or in this case about 70/2000 = 35 nm from the alkaline channel. The balance in flux at this point should equate to pH 7, therefore, there should be a steep pH gradient over approximately 35 nm, from pH 7 to 11 in the alkaline channel, or 3 pH units across 26 nm, assuming (most simply) a linear change in concentration. If the length of a single mackinawite nanocrystal is about 20 nm, then it is feasible that prebiotic vectorial chemistry could indeed support gradients of approximately 3 pH units across single nanocrystals, potentially facilitating the reduction of CO_2_ as discussed above. But even if true, this value is less than the required steepness of gradient needed to reduce CO_2_, assuming H_2_ saturation at atmospheric concentration. As noted earlier, the Δ*G^o^'* for the CH_2_O/HCOO^–^ couple is +38 kJ mol^−1^. A gradient of 5 pH units across a single mackinawite crystal would render the reduction exergonic because Δ*G* = –*nF* Δ*E_h_*, in which *n* is the number of electrons, *F* is the Faraday constant and Δ*E_h_* is the redox potential difference at 0.058 V per pH unit. In this case, Δ*G* *=* –2 × 96.5 × 0.290 *V* = –56 kJ mol^−1^, or an overall Δ*G'* of +38–56 = –18 kJ mol^−1^. But with a smaller 3-pH unit gradient across a single nanocrystal, Δ*G* *=* –2 × 96.5 × 0.174 V = –34 kJ mol^−1^, giving an overall Δ*G'* of +38–34 = +4 kJ mol^−1^, which thermodynamically could no longer drive the reduction of CO_2_, although it is on the point of doing so given the margin for uncertainty.

The reduction of CO_2_ by H_2_ depends not only on the redox potential but also on the concentration of the gases involved. A problem with a continuous-flow microfluidic chip is that H_2_ is poorly soluble at atmospheric pressure, saturating at 0.78 mM, which falls further at 70°C to 0.55 mM. The solubility of CO_2_ declines even more steeply with temperature, falling from 34 mM at 25°C to 11 mM at 70°C. However, the concentration of $\mathrm{HC}O_{3}^{\text{–}}$ added to our ocean solution was 10 mM, which at pH 6 should partition to 5 mM CO_2_, well below saturation at 70°C. Given an initial background contamination of CH_2_O of approximately 20 nM [95], Δ*G* *=* Δ*G^o^* + 2.3*RT* log_10_ [CH_2_O]/[H_2_]^2^[CO_2_], then Δ*G* = 4 + (5.698 × log_10_ 2 × 10^–8^/ 0.00055^2^ × 0.005) = +10 kJ mol^−1^, so again moderately endergonic (this assumes water at unity and a requirement for 2H_2_ to form CH_2_O + H_2_O). Only if the pH gradients were greatly steeper than those estimated here could CO_2_ reduction take place. For example, if the thinnest section of the barrier was approximately 30 µm thick, giving a pH gradient of 4 pH units across approximately 15 nm, the Δ*G'* would be just sufficient to drive CO_2_ reduction. The calculations described here, therefore, explain the difficulty of reducing CO_2_ in a microfluidic reactor running at atmospheric pressure, and this is consistent with our failure to reproducibly detect organics in the reactor to date despite some success (S Lim, N Lane 2016, unpublished observations) [62]. Others have also struggled to synthesize organics from H_2_ and CO_2_ in microfluidic reactors [76]. However, at higher pressures the saturation of H_2_ increases. At Lost City, the H_2_ concentration is approximately 15 mM [96,97], which gives a Δ*G* = 4 + (5.698 × log_10_ 2 × 10^–8^/0.015^2^ × 0.005) = –6 kJ mol^−1^, favouring CO_2_ reduction. If H_2_ concentration approaches 200 mM, as reported for the formation of minerals such as awaruite in hydrothermal conditions [98–100] then Δ*G* = –20 kJ mol^−1^ and CO_2_ reduction by H_2_ should proceed readily, given suitable catalysts. Other groups have also advocated the need for high-pressure to drive the reduction of CO_2_ by H_2_ [76] but to our knowledge this is the first study to constrain the dynamics of H^+^ gradients across Fe(Ni)S barriers in relation to CO_2_ reduction in a microfluidic reactor.

## 4. Conclusion

Phylogenetics and comparative physiology suggest that LUCA was an anaerobic autotroph that grew from the reaction between H_2_ and CO_2_ via some form of the acetyl CoA pathway [1,13–16,59] feeding into an incomplete reductive Krebs cycle [5,29,31]. However, while stronger electron donors such as Fe^0^ have been shown to reduce CO_2_ to carboxylic acids [26,27,29], the reaction between H_2_ and CO_2_ is difficult to achieve under prebiotic conditions at atmospheric pressure. Putatively ancestral autotrophs such as methanogens use a membrane-bound proton-motive Fe(Ni)S protein, the energy-converting hydrogenase, which uses the proton gradient to reduce ferredoxin and thence CO_2_ [7,8,48–50]. The transfer of protons across inorganic barriers containing Fe(Ni)S minerals prefigures both the magnitude and polarity of transmembrane electrochemical ion gradients in cells [41,58,59]. We have shown in a microfluidic chip that the permeability of disordered Fe(Ni)S barriers to H^+^ is about two million-fold greater than OH^–^, so steep pH gradients in the order of 3 pH units across 20–30 nm can exist across the barrier. These sharp H^+^ gradients could facilitate the reduction of CO_2_ by H_2_ across single mackinawite nanocrystals close to the alkaline face of the barrier. However, at atmospheric pressure, the low partial pressure of H_2_ means this reduction is borderline endergonic, and therefore unlikely to proceed. At partial pressures of H_2_ equivalent to those found in deep-sea hydrothermal systems the reaction should proceed exergonically. This warrants further experimentation in a high-pressure version of the microfluidic device presented here. The advantage of vectorial chemistry as a driving force for CO_2_ reduction at the origin of life is that it modulates the redox potential of H_2_, CO_2_ and Fe(Ni)S clusters, driving growth in a fashion that is topologically analogous and arguably homologous with that of cells [19,25,42,52,101]. Because membrane-associated Fe(Ni)S clusters are catalytic and self-assemble in association with fatty acid bilayers, they can in principle give rise to a form of membrane heredity that fosters growth and ultimately genetic heredity at the origin of life.

## Data accessibility

Additional data on the design, fabrication and operation of the microfluidic reactor are available from the authors.
